# Ultra-multiplex PCR technique to guide treatment of *Aspergillus*-infected aortic valve prostheses

**DOI:** 10.1515/biol-2022-0629

**Published:** 2023-07-07

**Authors:** Zhe Li, Da-Wei Li

**Affiliations:** Department of Intensive Care Unit, Sixth Medical Center of Chinese People’s Liberation Army General Hospital, No. 6, FuCheng Road, Haidian District, Beijing 100037, China

**Keywords:** ultra-multiplex polymer chain reaction, *Aspergillus* infection, aortic valve prostheses, infective endocarditis

## Abstract

Prosthetic valve endocarditis is a serious complication after heart valve replacement, accounting for about 20–30% of infective endocarditis (IE). Aspergillosis infection accounts for 25–30% of fungal endocarditis, and the mortality rate is 42–68%. *Aspergillus* IE often has negative blood cultures and lacks fever, which makes diagnosis difficult and delays antifungal therapy. Our study reported a case of IE in a patient with *Aspergillus* infection after aortic valve replacement. Ultra-multiplex polymerase chain reaction was used to identify *Aspergillus* infection and guide treatment. The purpose of this study was to enhance the understanding of the management of patients with endocarditis infected by fungi after valve replacement regarding the early detection, timely intervention, and treatment of the fungal infection to reduce the risk of death and improve the long-term survival of patients.

## 1 Introduction

Infective endocarditis (IE) is a serious complication that increases the risk of early death for patients after heart valve replacement. Clinically, prosthetic valve infections account for about 20–30% of IE. The incidence after aortic valve replacement is approximately 0.57% per person per year, with the highest risk being within 1 year after surgery [1]. Fungal endocarditis is rare but often fatal. Traditional fungal culture of low sensitivity (63%) and lengthy culture process lead to a delay in obtaining clinical evidence of pathogenic microorganisms and consequently delays antifungal therapy [2,3]. The 2020 European Confederation of Medical Mycology consensus states that polymerase chain reaction (PCR) methods on blood and bronchoalveolar fluid provides a robust diagnostic test for screening and confirming the diagnosis of *Aspergillus* infection [4]. Our study reported a case of IE in a patient with *Aspergillus* infection after aortic valve replacement. Ultra-multiplex PCR was used to identify *Aspergillus* infection and guide treatment. The purpose of this study was to enhance the understanding of the management of patients with endocarditis infected by fungi after valve replacement, regarding the early detection, timely intervention, and treatment of the fungal infection to reduce the risk of death and improve the long-term survival of patients.

## 2 Background

### 2.1 General information

**Figure 1:**
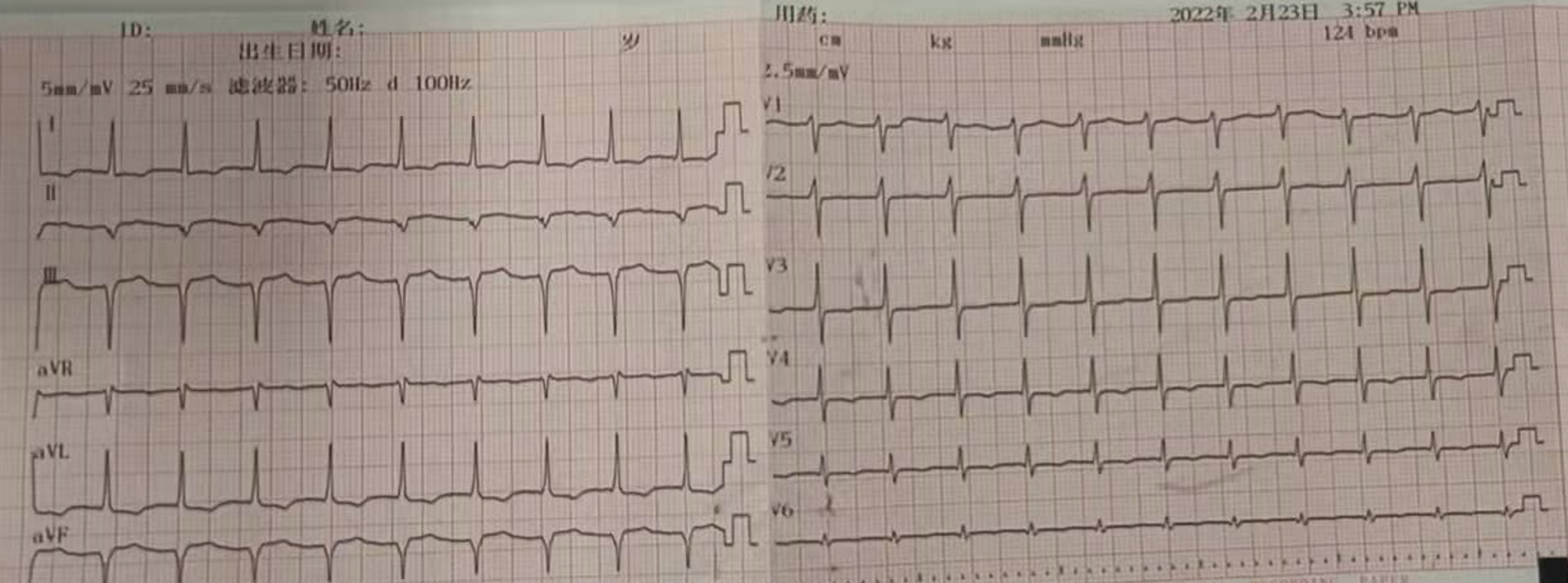
Electrocardiogram. Electrocardiogram showed low T wave, no ST-elevation or depression, and no dynamic development.

**Figure 2:**
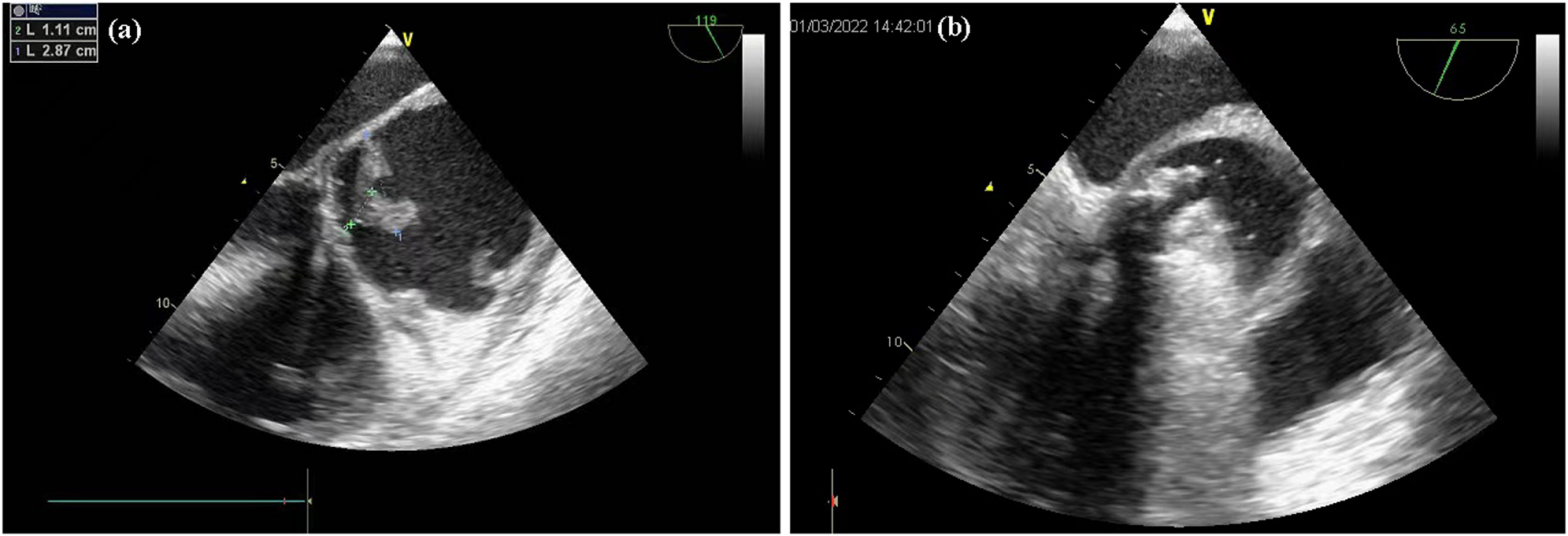
TEE. The picture shows the prosthetic aortic valve incompetence. Multiple vegetations are present in the valve frame ascending aortic wall, the largest vegetation is 28 mm × 11 mm with an obvious range of motion. There is an obvious dilation of the ascending aorta whose internal diameter is approximately 64 mm, accompanied by a thickening of the wall whose thickness is approximately 6.6 mm. There is mild regurgitation of the mitral and tricuspid valves. (a) TEE-long axis of the aorta, (b) TEE-short axis of the aorta.


The case concerns a 58-year-old male patient who underwent aortic valve replacement on December 17, 2021 for severe aortic valve insufficiency and received warfarin anticoagulation postoperatively. On January 7, 2022, the patient developed mobility impairment of the right limb. A cranial computed tomography (CT) scan showed cerebral infarction, so antiplatelet and anticoagulant therapy was administered. On January 12, the patient developed a fever with a maximum body temperature of 39.8°C. Transthoracic echocardiography (TTE) showed normal mechanical aortic valve function after mechanical aortic valve replacement. The patient presented an aortic root lesion (abscess ulceration with multiple superfluous formations and localized aortic root entrapment combined with an aneurysm) and segmental ventricular wall motion abnormalities. Intravenous daptomycin 500 mg was administered daily for anti-infection. The patient had recurrent fever with temperature fluctuations between 38–39°C. On February 20, the patient developed confusion, and a cranial CT scan showed a hematoma in the right frontal lobe, a subarachnoid hemorrhage in the right occipital lobe, and multiple lacunar infarctions in the bilateral basal ganglia and left paraventricular area. Pulmonary CT showed bronchitis, and 1 g of Tienam was given every 8 h in combination with 500 mg of daptomycin daily for anti-infection. On February 23, the patient developed chills and another high fever, with a maximum body temperature of 39°C, rapid breathing of 30–40 breaths per minute, and declining consciousness. For further treatment, he was transferred to our department on February 23. The patient had previous diabetes mellitus and pre-excitation syndrome and had undergone radiofrequency ablation. He entered our department with the test results as shown in Table 1. We were also provided with an electrocardiogram (Figure 1) and a transesophageal echocardiogram (TEE; Figure 2a, b). The patient had been diagnosed with cerebral infarction, cerebral hemorrhage, myocardial infarction, pulmonary infection, aortic insufficiency, IE after heart valve replacement, multiple organ dysfunction syndromes, renal insufficiency, cardiac insufficiency, abnormal coagulation function, abnormal liver function, type 2 diabetes, and anemia.

**Table 1:** Comparison of laboratory tests before and after treatment

Test	Prior treatment	After treatment
Leukocyte count (×10^9^/L)	18.31	11.63
Neutrophils (%)	87.5	79.9
Hemoglobin (g/L)	75	76
Platelets (×10^9^/L)	263	202
Alanine aminotransferase (U/L)	58.7	24.9
Serum creatinine (µmol/L)	327.6	254.8
Creatine kinase-MB isoenzyme (ng/mL)	4.0	<2
Troponin I (ng/mL)	11	7.8
Myoglobin (ng/mL)	44	352
NT-proBNP (ng/L)	35,000	17,100
D-dimer (ng/mL)	17,000	47,000
Procalcitonin (ng/mL)	3.86	1.29
β-d-glucan (pg/mL)	343.84	270.87
Galactomannan	0.83	1.04
C-reactive protein (mg/mL)	240.2	165.45
Interleukin-6 (pg/mL)	137.8	85.12

**Informed consent:** Informed consent has been obtained from all individuals included in this study.
**Ethical approval:** The research related to human use has been complied with all the relevant national regulations, institutional policies and in accordance with the tenets of the Helsinki Declaration, and has been approved by Ethics Committee of Sixth Medical Center of Chinese People’s Liberation Army General Hospital.

### 2.2 Treatment situation

**Figure 3:**
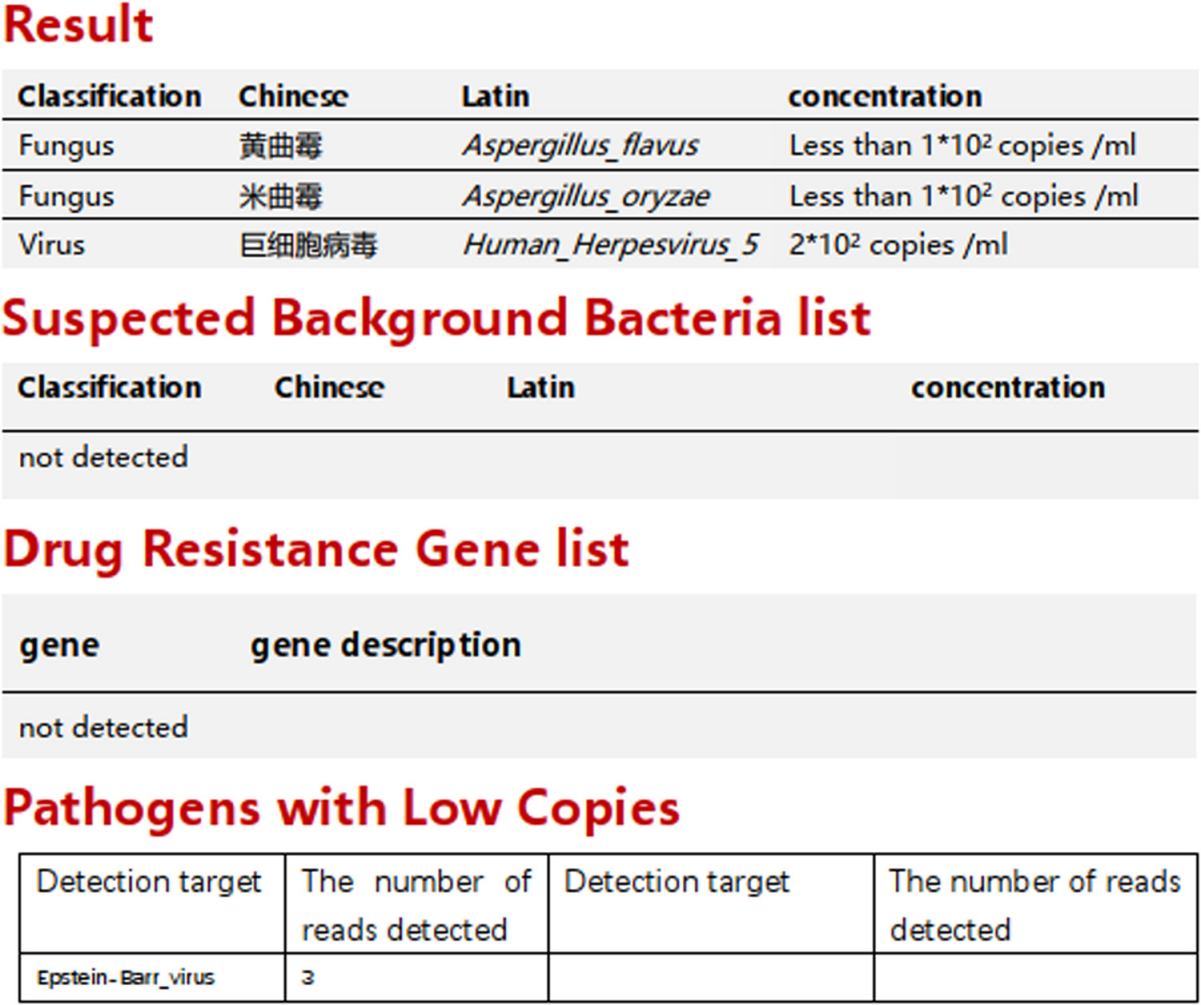
The result of ultra-multiplex PCR using blood.

The patient was admitted with respiratory distress, a Glasgow Coma Scale of less than 8, and intermittent convulsions. A ventilator with tracheal intubation was used to assist respiration, and sedatives and sodium valproate were pumped to control epilepsy. Bronchoscopy showed normal mucosa of both bronchi, and a small amount of thin yellow-white sputum was visible at the left and right bronchial openings. Daptomycin and Tienam continued to be given to fight infection. On March 24, blood was simultaneously collected for culture and testing and sent to the hospital for inspection by ultra-multiplex PCR. The multiplex PCR results received on February 25 were as follows (Figure 3): *Aspergillus flavus* <1 × 10^2^/L, *Aspergillus oryzae* <1 × 10^2^/L, *cytomegalovirus* 2 × 10^2^/L, *G* test 343.84 pg/mL, and GM 0.83. Test results indicated that the patient had endocarditis infected with Aspergillus, and the first dose of voriconazole was added at 6 mg/kg twice per day and was administered by 2 mg/day intravenous infusions maintained at 4 mg/kg. On February 28, the patient’s body temperature dropped to 37.8°C, and the test results were as shown in Table 1: Blood culture results reported negative on March 2.

**Figure 4:**
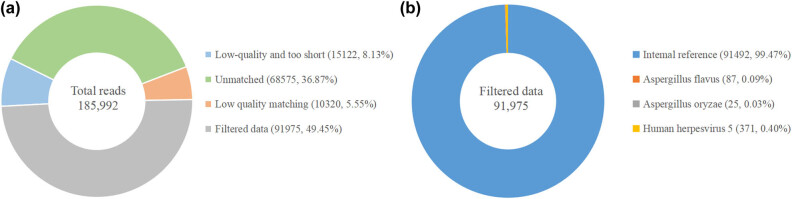
The results of ptNGS. (a) 185,992 is the total series of measurements. After eliminating the number of short sequences with low quality, unmatched sequences and matched sequences with low quality, the remaining number of valid sequences is 91,975, (b) In this 91,975, the number of effective sequences filtered was 91,492, the number of Aflatus sequences was 87, the number of Aspergillus sequences was 25, and the number of human herpesvirus type 5 sequences was 371.

*Aspergillus flavus* and *Aspergillus oryzae* were detected by pathogen-targeted metagenomics next-generation sequencing (ptNGS) of the blood (87 and 25 Copies/mL), Table 2 and Figure 4a, b. The primer sequences are as shown in Table 3.

**Table 2:** Microbes detected by ptNGS

Parameters	Microbe	Copies/mL
Pathogenic microorganisms	*Aspergillus flavus*	87
	*Aspergillus oryzae*	25
Background microorganisms	Human herpesvirus 5	317

ptNGS: pathogen-targeted next-generation sequencing.

**Table 3:** Primer sequences of *Aspergillus flavus/Aspergillus oryzae*

*Aspergillus flavus* primer sequences		
Primer	F	R
	GTCTTCCGATCGGAATCGTG	CTGGTTGCGTTATAAATACTGGCC
	TGGCCCTTCTCAACCTCACT	CGTCGCAAGTGAAACTCCAA
	GTTCCGTTGGAGCTGTCGATATA	CGTATCTACTTCCTCAAGCGCTACT
	CTACCTTCTCCGAAATGGCTTG	CTAGAGGGCAATAGTGGAGTTCAAG

*Aspergillus oryzae* primer sequences		
Primer	F	R
	TACTTTCTTTGGGGGTTTGTCTGGT	ACTCCAAGCCAGTTATTTCACATCAC

Our treatment adjustment for this patient was to report the ultra-multiplex PCR results before obtaining a negative blood culture result for 5 days and to adjust the antibiotic time schedule, which led to effective infection control, successful ventilator withdrawal, and a clear state of consciousness. The patient was subsequently transferred out of the intensive care unit. Unfortunately, in the later stage, a massive cerebral hemorrhage combined with a new cerebral infarction led to the death of the patient.

## 3 Discussion

With the increase in patients undergoing valve replacement, prosthetic valve endocarditis (PVE) has become an infectious disease of serious concern. Endocarditis is contracted by 1–6% of patients who have received a prosthetic valve replacement, accounting for 20–30% of all IE [5]. The annual incidence of IE for valve replacement patients is 0.3–1.2% [6,7], with aortic and mitral valves having approximately the same probability of PVE [8,9]. Without early recognition and treatment, the expected mortality rate is high. Even with timely diagnosis, antibiotic treatment, and valve replacement, the mortality rate ranges from 26 to 75% in medically treated patients vs 23–43% in surgically treated patients [10].

The onset of IE after valve replacement may occur early or later but is usually within 12 months. The timing of infection reflects different pathogenic mechanisms. Generally, PVE infection (less than 12 months postoperation) is mostly caused by microorganisms reaching the prosthetic valve either through direct intraoperative contamination or hematogenous spread in the first few days or weeks after surgery. In the early stage after valve implantation, microorganisms can enter the paravalvular tissue along the suture route and become encapsulated by host proteins such as fibrin, thereby, causing infection of the valve and surrounding tissue. This is why early paravalvular abscesses are particularly common. Twelve-month postoperative valve infection is caused by the formation of microthrombi (composed of platelets and fibrin) due to structural changes in the heart that facilitate the attachment of microorganisms, leading to infection [11]. With the passage of postoperation time, the endothelialization of paravalvular tissue can protect paravalvular tissue from infection. Therefore, paravalvular tissue is less likely to affect advanced PVE unless the pathogen causing the infection is *Staphylococcus aureus* or another highly virulent or invasive pathogen [12]. Therefore, late-onset infections are rarely complicated by paravalvular abscesses or valve dehiscence and are usually limited to the suture ring or prosthetic valve. The risk of PVE is greatest in the first year after implantation, in the first 6 months postoperatively at 1.4–3.1%, and lower thereafter, but consistently at 0.2–0.35% per patient per year. The risk of infection is higher for mechanical valves than for biological valves in the first 3 months and is the same for both valves after 5 years [1].

The pathogenic microorganisms of early PVE infection are different from that of natural valve endocarditis. PVE is mostly related to perioperative infection or central venous catheter infection and is likely to be nosocomial. The most common pathogens causing PVE are *Staphylococcus aureus* and coagulase-negative staphylococci, followed by gram-negative bacilli and *Candida*. Fungal endocarditis is rare, accounting for 1–2% of all IE. *Candida* is the most common fungal pathogen causing PVE, accounting for 49.6% (*Candida albicans* 37% and *Candida parapsilosis* 31.5%) [13]. *Aspergillus* fungi account for 30% (*Aspergillus fumigatus* 66.7% and *Aspergillus flavus* 22.8%), *Scedosporium apiospermum* account for 3.2% [14].

After valve replacement, it is necessary to be alert for PVE in patients if they have nonspecific unexplained symptoms such as fever, chills, anorexia, weight loss, or repeated bacteremia. The diagnosis of PVE relies on clinical manifestations, blood cultures (or other microbiological evidence), and echocardiography. The diagnosis of fungal endocarditis is far more difficult than it is for bacterial endocarditis, mainly because of lack of symptoms such as fever, splenomegaly, digital clubbing, or typical immune response symptoms such as Roth spots or Osler nodules. Fungal vegetations tend to be large and brittle and slough off, causing a greater risk of embolism than bacterial endocarditis. Many patients with endocarditis infected by *Aspergillus fumigatus* are admitted to other departments because of the tendency to shed large vegetations that predispose them to cerebrovascular and lower extremity vascular embolism [15]. Meena et al. [14] conducted a systematic review of 250 cases of fungal endocarditis reported between January 2000 and December 2020. The median time for symptom onset was 14 days postoperation. On general examination, fever was the most common symptom (62.1%), followed by dyspnea/chest pain (37.1%), new heart murmurs (32.8%), and splenomegaly (10.3%). At the same time, complications were the first manifestation of fungal endocarditis, the most common being peripheral vascular embolism (34.5%) followed by stroke, congestive heart failure, and pulmonary embolism, 27.9, 17, and 12.5%, respectively.

All patients with suspected PVE should undergo cardiac ultrasonography. TTE reveals valve involvement and redundancy in the majority of patients and abnormalities in nearly 13.6% of patients without TTE [10]. Paravalvular abscesses and fissures are difficult to evaluate by TTE. In addition, prosthetic valves can produce humming and shadowing that can obscure key patient presentations [16]. TEE is recommended for every patient suspected of having prosthetic fungal endocarditis to improve diagnostic accuracy and detection time. If the initial TEE is negative or indeterminate and PVE is clinically suspected, TEE should be repeated 5–7 days later if not contraindicated.

In addition to echocardiography and other examinations, the diagnosis of fungal endocarditis likely requires blood or tissue culture and histopathological analysis. However, the culture time of fungi is long, with a median time of 7 days, and the positive rate of blood culture of fungi, especially *Aspergillus*, is low [17], which leads to delayed diagnosis and treatment and significantly reduces the survival rate of patients with fungi, especially with *Aspergillus* endocarditis. Meena and others have shown [12] that 33.9% of IE with negative blood culture is a fungal infection. However, the positive of blood cultures for *Aspergillus*-infected endocarditis is only 28%, compared with 88% for *Candida*-infected endocarditis. The rates of positive tissue cultures and histopathological examinations are higher at 91.1% but are limited by the inconvenience to physicians in obtaining clinical materials. There are many new methods for fungal diagnoses, such as the *G* Test, GM Test, PCR technology, and gene detection of second-generation sequencing. The positive rate of *G* is 88.9%, the sensitivity of galactomannan detection and mannan/anti-mannan antibody is 83%, and PCR primer has been used for molecular diagnosis with good sensitivity and specificity in previous studies [18,19].

The patient in our case, who suffered complications of thrombosis, fever, and cerebral hemorrhage after aortic valve replacement, was admitted to our hospital. Although neurologic complications are the most frequent extracardiac complications of left-sided IE occurring in 20–55% of patients, including acute ischemic stroke, cerebral microbleeds, cerebral abscess, mycotic aneurysms, and meningoencephalitis. And cryptogenic stroke can be the first sign of IE in about 35% of patients and is associated with increased morbidity and mortality [20]. However, due to the lacking data of other hospitals and surgical procedures, we do not have enough evidence to prove whether the patient’s cerebral infarction is related to vegetations. But fever of unknown origin after antibacterial treatment makes us confused, whether there is infection by other pathogens? Considering the low detection rate and long progress of pathogens associated with *Aspergillus* infection, ultra-multiplex PCR (ptNGS) was conducted.

Multiplex PCR was first introduced in 1988 when it was used to amplify the Duchenne muscular dystrophy gene to enable mutation detection. When the number of primer pairs in a single PCR exceeds 100, it is often referred to as ultra-multiplex PCR sequencing. By pre-designing thousands of targeted primers, ptNGS performs highly uniform ultra-multiplex PCR amplification and signal amplification for target-specific fragments in the sample of the same reaction system. And then, a large number of targets are enriched, amplification products are synchronously deep sequenced, non-target fragments are eliminated, and quantitative analysis is carried out by high-throughput sequencing. Therefore, this high-throughput ptNGS technology can quickly and economically exploit the dual advantages of the high sensitivity of targeted amplification and high specificity. Because the primer is pre-designed for the target, it is unaffected by the human genome and that of the background bacteria in the sample during the gene capture stage. Not only can the target gene of the pathogen in the sample be captured without interference, but there is an efficient collection of the pathogen’s nucleic acid, such as *Mycobacterium tuberculosis*, *Legionella*, *Brucella,* and others. At present, ultra-multiplex PCR technology has been widely used in various fields of molecular diagnosis, including the detection of monogenic hereditary diseases, the detection of tumor drug genes, polymorphic loci, and forensic medicine. Recently, ultra-multiplex PCR technology has also begun to emerge in the field of pathogenic microorganism detection. Shanghai Pulmonary Hospital can screen about 30 common tuberculosis *Mycobacterium* and non-tuberculous *Mycobacterium* species in a single test using multiplex PCR technology [21]. The Second Hospital of Hebei Medical University can use ultra-multiplex PCR technology to detect more than 300 common clinical pathogens in one reaction [22]. Compared with traditional single-tube PCR assays, these assays have the characteristics of high throughput, high efficiency, and cost-effectiveness. Compared with 7 days for fungal culture, the detection time of ultra-multiplex PCR is 1 day, a great benefit for the anti-infective treatment of critically ill patients.

For endocarditis caused by a fungal infection of prosthetic valves, the main treatment is a systemic application of antifungal drugs combined with surgery, the latter being more important. The operation time varies depending on the clinical situation, anatomical manifestations, and complications. Cases of ischemic and hemorrhagic brain injury are mainly treated by neurologists and neurosurgeons. New massive cerebral infarction or cerebral hemorrhage should be considered a possibility at least 4 weeks prior to valve replacement in cases of hemodynamic stability and low risk of embolic recurrence [1].

This patient was not suitable for surgical treatment because of serious complications, so we used voriconazole. Voriconazole is the first-line treatment for aspergillosis. With the treatment of voriconazole, the patient’s body temperature was normal, and the endotracheal tube was removed. However, the patient suffered more serious cerebral hemorrhage. Regardless of etiology, delays in diagnosis are common and associated with worse outcomes, for patients with diagnostic difficulties, early ultra-multiplex PCR (using biological samples with only a few pathogens) are helpful for early diagnosis and treatment, potentially allowing patients to achieve favorable outcomes.

## 4 Conclusion

PVE, especially from fungal infection, in valve replacement is a serious potential complication with high mortality. Slow diagnosis of fungal infection, especially in Aspergillus-infected PVE, leads to delayed antifungal treatment that increases the risk of serious complications and even death in patients. Therefore, it is necessary to be alert to any recurrent fever or unexplained embolism in patients after prosthetic valve replacement and alert to the possibility of prosthetic valve infection. Echocardiography remains the first step toward determining the cause of any symptoms, and TEE is recommended. In addition to improving the screening of routine infection indicators such as routine blood tests, procalcitonin tests, *G* tests, and GM tests, multiplex PCR combined with next-generation targeted sequencing technology can quickly obtain pathogenic evidence and increase the culture positivity rate, enabling the application of anti-infective drugs early to control infection and create better conditions for subsequent surgical treatment.
